# Effect of Different Polymer Modifiers on the Long-Term Rutting and Cracking Resistance of Asphalt Mixtures

**DOI:** 10.3390/ma14123359

**Published:** 2021-06-17

**Authors:** Bangwei Wu, Chufan Luo, Zhaohui Pei, Ji Xia, Chuangchuang Chen, Aihong Kang

**Affiliations:** 1 College of Civil Science and Engineering, Yangzhou University, Yangzhou 225127, China; wubw@yzu.edu.cn (B.W.); MZ220190387@yzu.edu.cn (C.L.); MZ220200335@yzu.edu.cn (Z.P.); MZ120200952@yzu.edu.cn (J.X.); MZ120200967@yzu.edu.cn (C.C.); 2 Research Center for Basalt Fiber Composite Construction Materials, Yangzhou University, Yangzhou 225127, China

**Keywords:** asphalt mixtures, polymer modifier, long-term performance, rutting resistance, cracking resistance

## Abstract

To evaluate the long-term performances of different polymer-modified asphalt mixtures, three modifiers were chosen to modify AC-13 (defined as the asphalt concrete with the aggregate nominal maximum particle size of 13.2 mm); namely, high viscosity modifier (HVM), high modulus modifier (HMM), and anti-rutting agent (ARA). The deformation and cracking resistance of different polymer-modified mixtures were checked at different aging conditions (unaged, short-term aged, and long-term aged for 5, 10, and 15 days respectively). The results of the Hamburg wheel-track test and uniaxial penetration test (UPT) showed that the rutting resistance of all asphalt mixtures changed in a V-shape as the aging progressed. From the unaged stage to the long-term aging stage (5 days), the rutting resistance decreases gradually. While after the long-term aging stage (5 days), the rutting resistance increases gradually. Results from the semicircular bending test (SCB) and the indirect tensile asphalt cracking test (IDEAL-CT) indicated that the cracking resistance of all the mixtures gradually decline with the deepening of the aging degree, indicating that aging weakens the crack resistance of asphalt mixtures. Additionally, test results showed that the rutting resistance of ARA AC-13 (defined as AC-13 containing ARA) is the best, the cracking resistances of ARA AC-13, HMM AC-13 (defined as AC-13 containing HMM) and HVM AC-13 (defined as AC-13 containing HVM) have no significant difference, and different polymer modifiers had different sensitivities to aging due to the polymer content and the type of modifier. The conclusions of this study help to further understand the long-term performance of polymer-modified asphalt mixtures during service life and to help guide the selection of modifiers for mixtures.

## 1. Introduction

Nowadays, polymer modifiers are widely used in pavement engineering to improve asphalt properties; styrene–butadiene–styrene (SBS) accounting for most of them. The results of a great deal of research have demonstrated that SBS can improve the rutting resistance, water stability, cracking resistance, and other properties of asphalt mixtures [1,2,3]. Research conducted on the National Center for Asphalt Technology (NCAT) test road compared the rutting resistance of SBS-modified asphalt mixture and unmodified asphalt mixture. The results showed that after nine million equivalent single axle loads, the rutting depths of the modified asphalt mix and the unmodified asphalt mix were 2.7 mm and 6 mm, respectively [4]. In addition to SBS, a number of other polymer modifiers are used in asphalt mixtures, such as polyethylene (PE) [5], ethylene vinyl acetate copolymer (EVA) [6], styrene butadiene rubber (SBR) [7], and so on; however, these modifiers are not widely used. In recent years, many other new types of modifiers have been put into use, such as anti-rutting agents (ARA), high modulus modifiers (HMM), high viscosity modifiers (HVM), etc. These have received more and more attention over time.

High modulus binders are widely used in several European countries, as well as China, to increase rutting resistance. Wu [8] investigated the high-temperature stability of high-modulus asphalt concrete, using the rutting test and footprint model test. The results showed that high-modulus asphalt mixtures have great resistance to deformation compared to ordinary asphalt mixtures. Lee et al. [9] used high modulus modifiers and SBS modifiers to prepare high modulus asphalt mixtures. They found a significant increase in the stiffness modulus of mixtures; crack resistance was not significantly reduced compared to unmodified asphalt mixtures. ARA is also widely used to improve the deformation resistance of asphalt mixtures. Ulucayli [10] concluded that the use of ARA can significantly improve the deformation resistance of asphalt mixtures. Chen [11] argued that ARA acts as an elastic particle embedded in the voids of an aggregate, which can reduce the void ratio of mixtures. Sun [12] investigated the performance of ARA-modified asphalt mixtures and concluded that the elastic component of ARA could reduce the rutting deformation of asphalt pavements. HVM is also a widely used asphalt mixture additive. Yang [13] argued that this modifier resulted in asphalt pavements with good resistance to cracking and deformation. Li [14] argued that HVM-modified asphalt helps to prevent short-term loosening of porous asphalt pavements.

With the increase in traffic load, there is a growing concern about the durability of asphalt mixtures. Thus, some researchers have used multiple polymers to enhance pavement durability. For example, Yuan [15] used “SBS+ARA” to improve the high-temperature performance of asphalt mixtures. In addition to using multiple polymers, the anti-aging properties of asphalt binders also play an important role in affecting pavement durability. During the service life of the asphalt pavement, moisture, oxygen, ultraviolet light, etc., will cause changes in the physical and chemical properties of asphalt [16,17]; that is, the aging of asphalt, which will ultimately have an impact on the asphalt mixture performance. Zhang [18] studied the influence of aging on the thermal behavior of SBR+SBS compound-modified asphalt. Additionally, some researchers studied the aging of polymer-modified asphalt to characterize the durability of mixtures. Cortizo [19] reported the thermal degradation of SBS during the aging of modified asphalt binder. Sugano [20] insisted that the degradation of SBS hurt binder performance. Zhao [21] explored the aging mechanism of SBS polymer-modified asphalt using its Fourier transform infrared spectrum and found that the chemical structure of SBS changed over the aging process. Yan [22] proposed an infrared index to quantify the extent of aging of SBS-modified asphalt. Margaritis [23] evaluated the feasibility of incorporating reclaimed polymer-modified binder in new surface layer mixtures.

It can be seen that a great deal of research has been conducted on polymer modifiers, and some useful conclusions have been drawn; however, most of the research focuses on only one kind of modifier, lacking a comprehensive comparison between different modifiers. In particular, the long-term performance of asphalt mixtures using different modifiers has not been adequately compared. Thus, the objective of this study is to evaluate the effects of different modifiers on the long-term performance of asphalt mixtures. Three polymer-modified asphalt mixes were aged for different times in a laboratory to simulate the different service times of the asphalt mixes in the field. Then, a series of tests was conducted to check the deformation and cracking performance of mixtures under different aging degrees.

## 2. Test Materials and Mixture Design

### 2.1. Asphalt Binder

SBS-modified asphalt was chosen as the control binder, and its basic properties are presented in Table 1. The asphalt properties meet the specification requirements.

### 2.2. Polymer Additives

**Figure 1:**
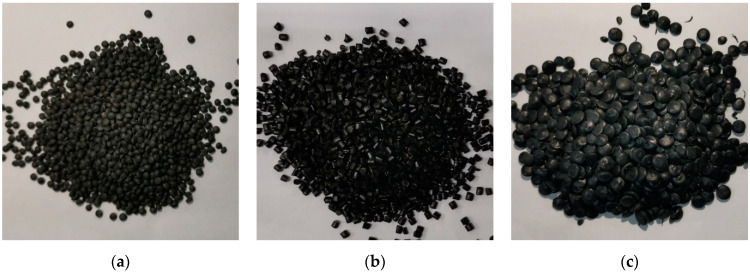
The appearance of the three modifiers: (**a**) HVM, (**b**) HMM, and (**c**) ARA.

Three modifier additives were chosen for this study, namely HVM, HMM, and ARA. The three modifiers were provided by WanPu Transportation Technology Co., Ltd., Jiangsu, China. The appearances of the three modifier additives are shown in Figure 1, and their basic properties are shown in Table 2.

**Figure 2:**
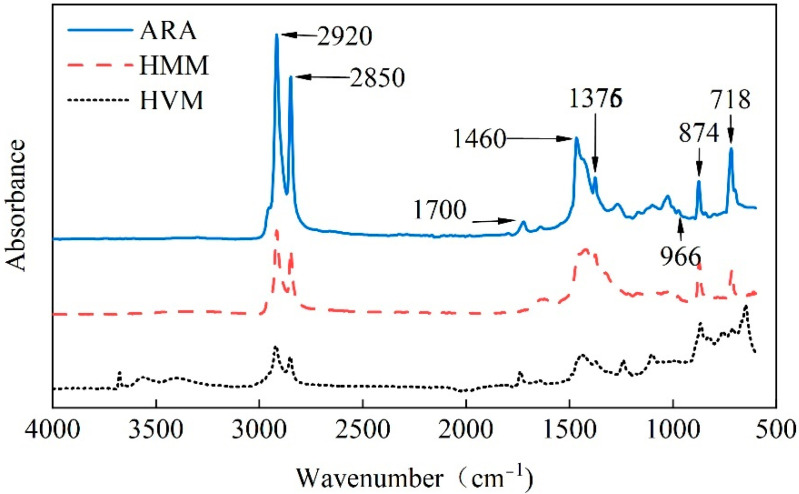
FTIR spectra of different modifiers.

In this research, an attenuated total internal reflectance Fourier transform infrared spectrometer (ATR-FTIR) was used to analyze the chemical compositions of the different modifiers, and the results are shown in Figure 2.

According to Figure 2, the characteristic peaks of the modifiers are similar. They are mainly concentrated at 2920 cm^−1^, 2850 cm^−1^, 1700 cm^−1^, 1460 cm^−1^, 1375 cm^−1^, 966 cm^−1^, and 718 cm^−1^. Although the graph shows that the characteristic peaks of the modifiers are very similar, each modifier has its own unique characteristic peaks. For example, ARA has more and smaller characteristic peaks than HMM and HVM. Additionally, the peak heights and peak areas of the three modifiers are quite different. This is mainly due to the different contents of alkanes, cycloalkanes, and other components. These differences may cause their different sensitivities to aging.

### 2.3. Aggregates

Limestone in four sizes, 10–15 mm, 5–10 mm, 3–5 mm, and 0–3 mm, was used for the asphalt mixture design. The filler was limestone powder. The basic properties of the aggregates are shown in Table 3. All the mechanical properties of the aggregates met the current specifications, namely the *Technical Specification for Construction of Highway Asphalt Pavements (JTG F40-2004)* [24].

### 2.4. Asphalt Mixture Design

**Figure 3:**
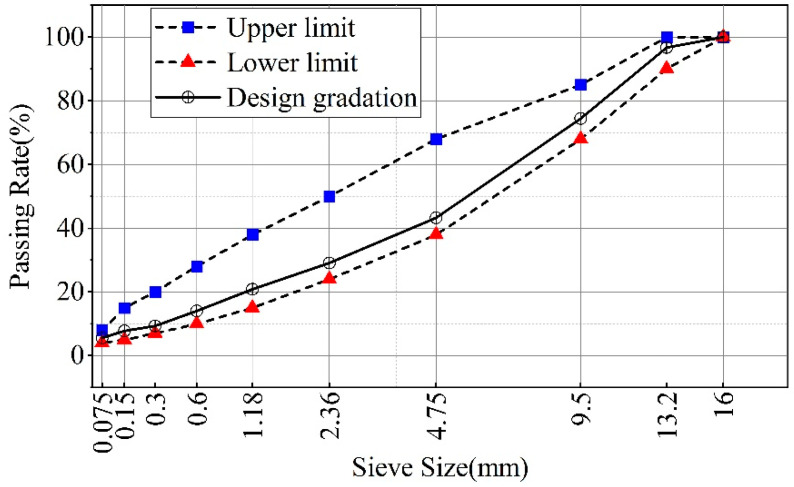
Design gradation of AC-13.

AC-13 was selected for this study. It is a widely used dense gradation type from China, and its nominal maximum aggregate size is 13.2 mm. AC-13 was designed using the Marshall method. The design gradation of AC-13 is shown in Figure 3. The volume parameters of AC-13 are shown in Table 4.

A total of four types of AC-13, namely SBS AC-13, SBS+HVM AC-13, SBS+HMM AC-13, and SBS+ARA AC-13, were prepared for the tests. The dosage of the modifier additive used in asphalt mixtures was 8% of the mass of SBS asphalt. Such a dosage is quite common in Chinese pavement projects.

In terms of AC-13 using modifier additives, they were fabricated using a “dry mixing” process to simulate the actual production process, which meant that the modifiers were first mixed and blended with aggregates for 90 s before adding the asphalt.

## 3. Methods

### 3.1. Aging Method

Asphalt mixtures were aged for different times to simulate different service life times of asphalt pavements. According to AASHTO R30 [25], short-term aged mixtures were used to simulate asphalt mixtures after being paved and long-term aged mixtures were prepared to simulate asphalt mixtures that had been paved five to seven years prior. The aging steps were conducted according to AASHTO R30. In the short-term aging process, the loose mixtures were paved evenly in a plate and put into an oven for 4 h at 135 °C (under forced ventilation); this needed to be stirred once an hour during this process. Then the loose mixtures was used to make the short-term aged asphalt mixtures. In the long-term aging process, the short-term aged mixture specimen was put into an oven for 5 days at 85 °C (under forced ventilation) to prepare the long-term aged asphalt mixture samples. 

Considering the difference in climate and traffic load between China and the United States, this study added two long-term-plus aging conditions, with extended aging times lasting for 10 and 15 days, respectively.

### 3.2. Test Methods for Deformation Property

#### 3.2.1. Uniaxial Penetration Test

**Figure 4:**
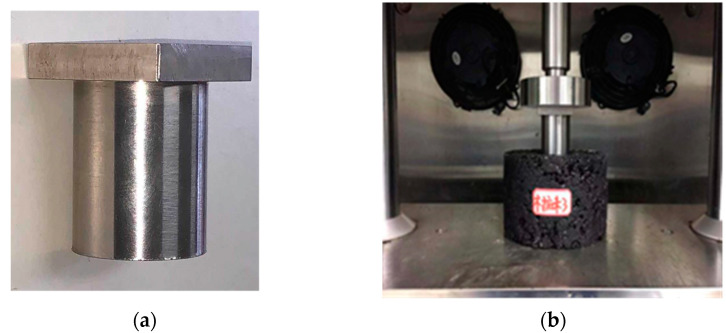
Images of the uniaxial penetration test: (**a**) steel plunger and (**b**) test process.

The uniaxial penetration test (shown in Figure 4) is a popular method to evaluate the shear strength of asphalt mixtures in China and was proposed by Sun in 2004 [26]. The diameter and height of the specimen were 150 mm and 100 mm, respectively. The specimen was loaded using a steel plunger at a speed of 1 mm/min. The diameter of the steel plunger was 42 mm and the test temperature was 60 °C. Shear strength can be expressed using Equation (1). Four specimens were taken for each test.

(1) $$\tau_{0}=f\times F/A_{c}$$

where *τ*_0_ is the shear strength (MPa), *F* is the maximum load (N), *A_c_* is the cross-section area of the steel plunger (mm^2^), and *f* is the shear coefficient, taken as 0.350.

#### 3.2.2. Hamburg Wheel-Tracking Test

The Hamburg wheel-tracking test was carried out using the AASHTO T324 specifications [27]. This test is used to determine the rutting property and moisture susceptibility of asphalt mixtures. The diameter and height of the specimen were 150 mm and 62 mm, respectively. The specimens were submerged in a 60 °C water bath, and a steel wheel made 52 ± 2 passes across the specimen per minute. The test was automatically stopped when a specimen had a rutting depth of 20 mm, or after 20,000 passes. Rutting depth and the stripping inflection point (SIP) were reported as test indicators.

### 3.3. Test Methods for Cracking Property

#### 3.3.1. Semicircular Bending Test

The SCB test was conducted according to AASHTO TP 124 [28]. This test was designed according to the Fracture mechanics theory. A cross section of the specimen was semi-circular-shaped (radius was 75 mm), and the specimen was pre-cut at a certain length, called the pre-cut length (pre-cut length was 20 mm). The difference between the radius of the specimen and the pre-cut length is called the ductile zone length (DZL). *G_f_* is the fracture energy and it was calculated according to Equation (2), where *W_f_* is the integral of the load–displacement curve and *Area_lig_* is the ligament area and the thickness of the specimen (*t* = 50 mm). Flexibility index (FI) was adopted to reflect the crack propagation rate and it was calculated using Equation (3), where |m| is the absolute value of the slope at the inflection point after the peak of the loading value. The FI value is negatively correlated with the crack propagation rate. Four specimens were used to determine the FI.

(2) $$G_{f}=\frac{W_{f}}{Area_{lig}}\times 10^{6}$$

(3) $$FI=\frac{G_{f}}{\left|m\right|}\times 0.01$$

#### 3.3.2. IDEAL-CT

The indirect tensile asphalt cracking test (IDEAL-CT) is similar to the traditional indirect tensile strength test. It was developed by Zhou [29] to evaluate the cracking propagation performance of asphalt mixtures. Cylindrical specimens with a diameter of 150 mm and a thickness of 62 mm were used in this study; the test was run at 25 °C. *CT_index_* is proposed to determine the crack growth rate of asphalt mixtures, which was defined using Equation (4). Four specimens were used for each test.

(4) $$CT_{index}=\frac{G_{f}}{\left|m_{75}\right|}\times \frac{l_{75}}{D}$$

where *G_f_* is the fracture energy (J/m^2^) and |*m*_75_| is the absolute value of the slope of the load–displacement curve at the point where the load is reduced to 75% of the peak load. *l*_75_ is the displacement at the point where the load is reduced to 75% of the peak load (mm), and *D* is the specimen diameter (mm). 

## 4. Results and Discussion

### 4.1. Effect of Modifiers on Deformation Performance of Asphalt Mixtures

#### 4.1.1. Uniaxial Penetration Test Results

**Figure 5:**
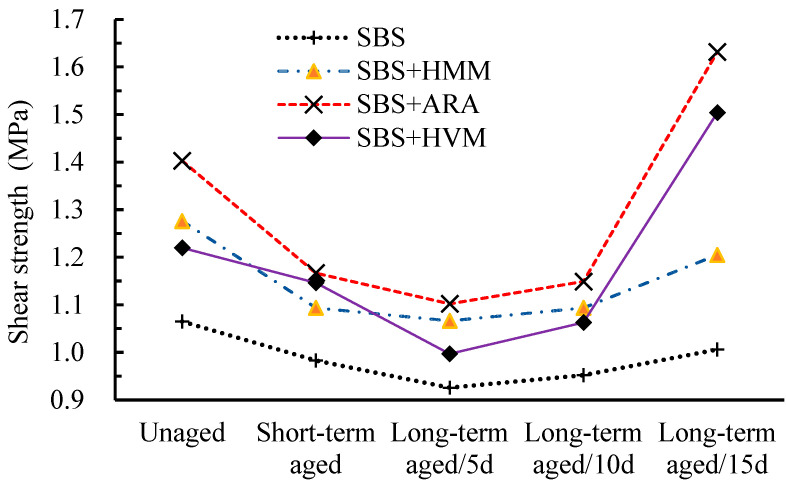
UPT results.

UPT test results are shown in Figure 5.

Some points can be observed from Figure 5.

(1) All the three modifier additives improved the shear strength of SBS AC-13, and the improvement varied at different aging stages of the asphalt mixture. For instance, at the unaged stage, HMM, ARA, and HVM improved the shear strength by up to 19.8%, 31.7%, and 14.5%, respectively. At the short-term aging stage, however, the corresponding improvements were 11.3%, 18.7%, and 16.6%, respectively. Additionally, ARA improved the shear strength of AC-13 the most in all the aging stages. At the short-term aging stage and long-term aging (15 days) stage, HMM increased the shear strength more than HVM; in other aging states, however, the situation was reversed. This phenomenon indicated that the sensitivity of different modifiers to the aging process was different.
(2) The shear strength of all asphalt mixtures changed into a V-shape as the aging progressed. From the unaged stage to the long-term aging stage (5 days), the shear strength decreased gradually. After the long-term aging stage (5 days), the shear strength increased gradually. Taking SBS AC13 as an example, its shear strength decreased by 7.7% and 13.1% after short-term aging and long-term aging, respectively, compared to the shear strength in the unaged stage. In contrast, based on the short-term aging shear strength, the 10-day-long-term aging and the 15-day-long-term aging performances increased by 2.8% and 8.6%, respectively. In addition, the shear strength of each asphalt mixture did not decrease or increase to the same extent, which further indicated that different modifiers have different sensitivities to aging, but they all have the lowest shear strength at the 5-day-long-term stage.

For unmodified pure asphalt, the more severe the aging, the harder the asphalt and the higher the corresponding shear strength became, but this was not the case for polymer-modified asphalt. For the aging of polymer-modified asphalt, there are two phenomena involved in the aging process [30,31,32]. One is the degradation of the modifier additive, causing the properties of SBS-modified asphalt to approach those of pure asphalt. The other is the aging of the pure asphalt. The former makes the binder softer, while the latter makes the binder stiffer. The actual shear strength of the asphalt mixture was determined according to the joint effect of these two phenomena. From Figure 5, it is implied that, before the long-term aging stage (5 days), polymer degradation dominates, while after the stage, pure asphalt aging dominates, eventually leading to a V-shaped change in the shear strength of the asphalt mixtures. In addition, the difference in the polymer content and type in HMM, HVM, and ARA lead to their different sensitivities to the aging process.

#### 4.1.2. Hamburg Wheel-Tracking Test Results

**Figure 6:**
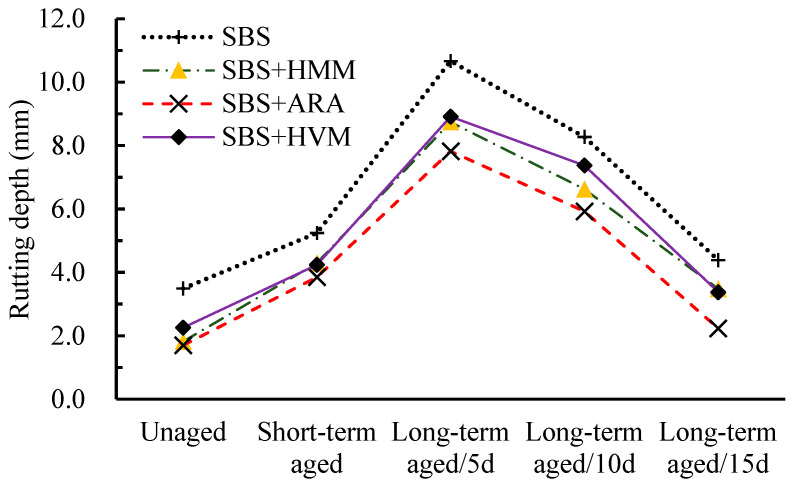
Rutting depth of different modified mixtures under different aging conditions.

**Figure 7:**
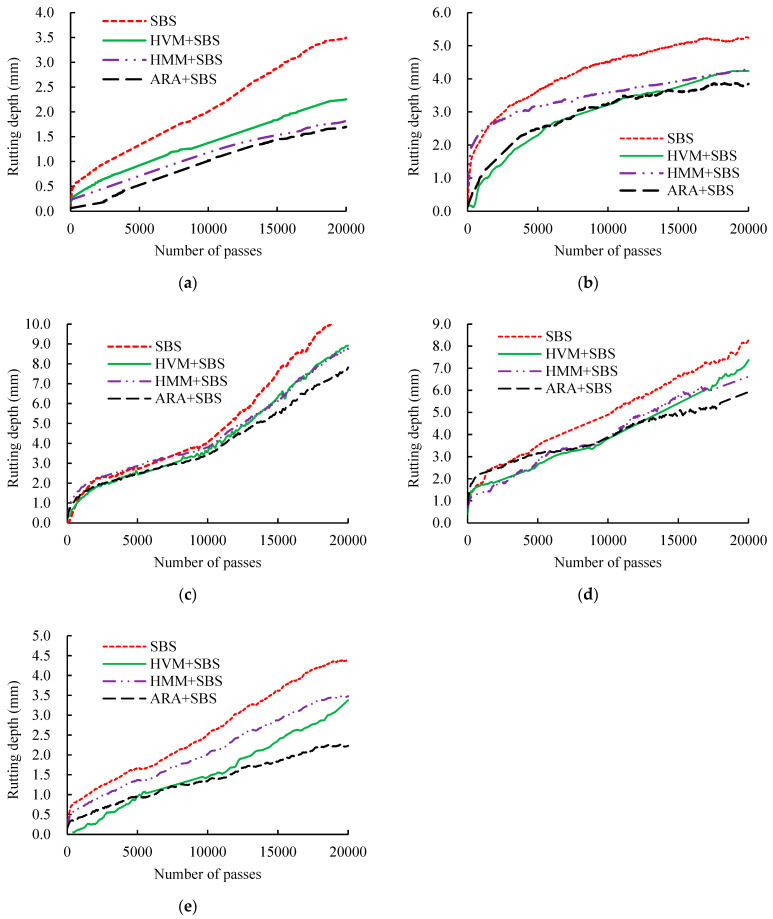
Deformation curve: (**a**) unaged stage, (**b**) short-term aging stage, (**c**) 5-day-long-term aging stage, (**d**) 10-day-long-term aging stage, (**e**) 15-day-long-term aging stage.

The results of Hamburg wheel-tracking test are shown in Figure 6 and Figure 7. Figure 6 presents the rutting depth of the mixtures under different aging conditions. Figure 7 shows the deformation curve during the tests.

It can be seen from Figure 6 that the rutting depth of all asphalt mixtures rose and then fell as the aging degree increased, and the largest rutting depth occurred after 5 days of long-term aging. This pattern is in full agreement with the results of the UPT test. Additionally, it can be observed from the deformation curves (seen in Figure 7) that SIP only occurred in the curve of the 5-day long-term aging, further demonstrating that the rutting resistances of mixtures for long-term aging (5 days) were the weakest. 

Another point from Figure 6 is that aging has an important effect on the rutting depth of mixtures. The rutting depth of asphalt mixtures can vary by several fold at different aging stages. Taking SBS AC-13 as an example, from the unaged stage to the long-term aging stage (15 days), the greatest rutting depth was about 11 mm, while the smallest rutting depth was only about 3.5 mm. Therefore, when polymer-modified asphalt mixtures are used for projects, attention must be paid to the changes in the rutting resistance of the mixtures due to aging.

In addition to this, it can also be seen from Figure 6 that all the three modifier additives improve the rutting resistance of AC-13, and the improvement from ARA was the most significant at any aging stage; the rutting resistances of HVM AC-13 and HMM AC-13 were different at different aging stages. For the short-term aging stage and long-term aging (15 days) stage, HVM AC-13 had a smaller rutting depth than HMM AC-13. In other aging states, however, the situation is reversed. This phenomenon is in full agreement with the UPT results.

### 4.2. Effect of Modifiers on Cracking Resistance of Asphalt Mixtures

**Figure 8:**
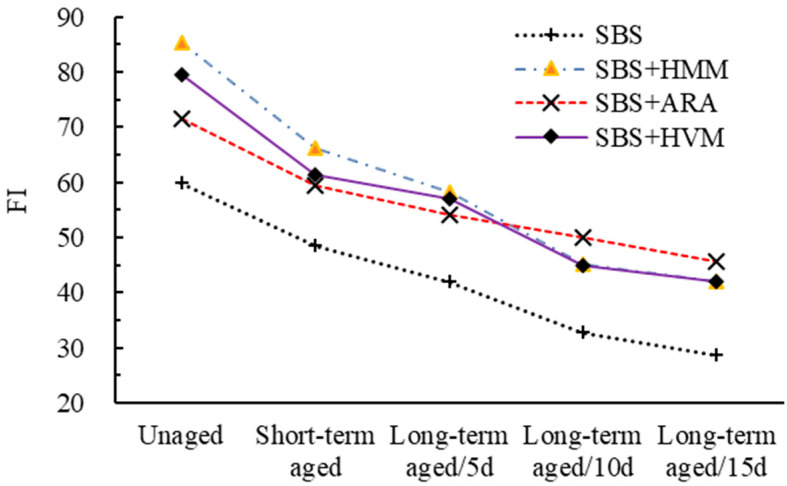
SCB results.

**Figure 9:**
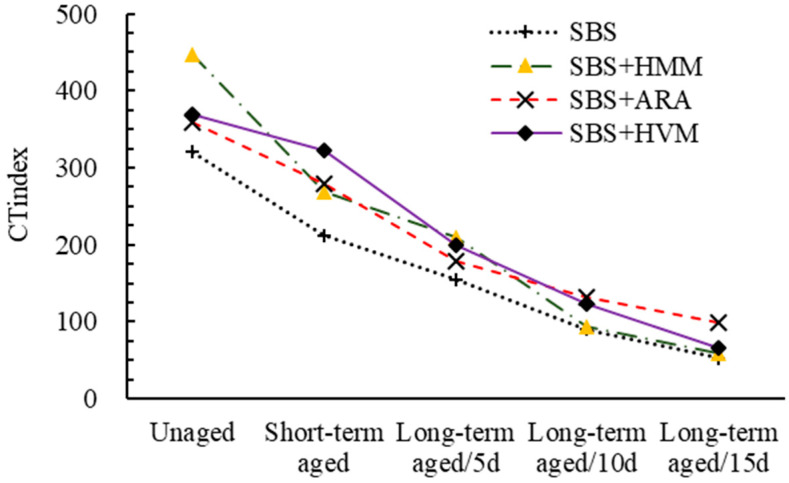
Result of IDEAL cracking tests.

The results of SCB and IDEAL-CT are shown in Figure 8 and Figure 9, respectively.

Both FI and *CT_index_* are indicators derived from fracture mechanics to characterize the crack extension properties of asphalt mixtures. From Figure 8 and Figure 9, it can be seen that the effect of aging on the crack resistance of different modified asphalt mixtures has roughly the same pattern. That is, with the increasing of the aging degree, both FI and *CT_index_* gradually decline. Taking ARA AC-13 as an example, compared to the FI in the unaged stage, the FI in the short-term aged stage and long-term ages (5 days, 10 days, and 15 days) stage decreased by 16.9%, 24.2%, 29.9%, and 36.1%, respectively. Thus, it can be concluded that the more severe the aging of the asphalt mixtures, the worse its anti-cracking properties. As analyzed earlier, there are two phenomena in the aging process of polymer-modified asphalt: one is the degradation of the modifier additive, the other is the aging of the pure asphalt. Both phenomena lead to asphalt hardening and cause a decrease in the crack resistance of the asphalt mixture.

In addition to this, it can also be observed that all the three modifier additives improve the cracking resistance of asphalt mixtures. However, as different modifiers are not equally sensitive to aging, this resulted in different modified asphalt mixtures exhibiting different crack resistances at different aging stages. For example, HMM AC-13 had the greatest *CT_index_* in the unaged state, while for the long-term aging state (15 days), the *CT_index_* of ARA AC-13 was the greatest.

**Figure 10:**
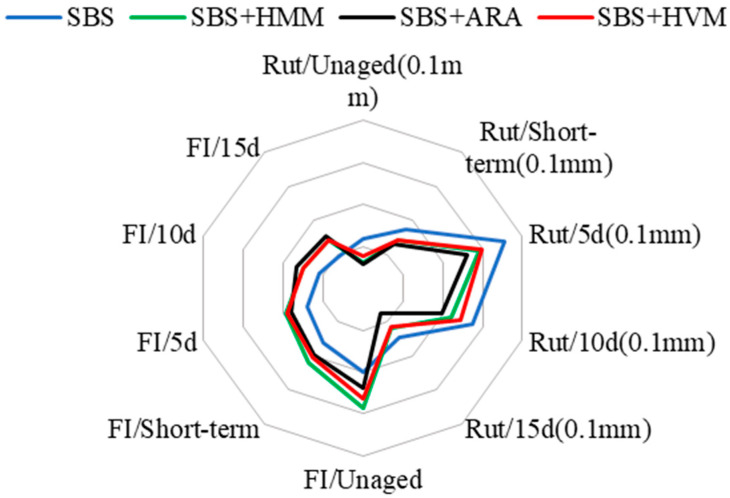
Performances of different polymer-modified asphalt mixtures.

Figure 10 comprehensively presents a comparison of the high temperature and cracking resistance of different modified asphalt mixes.

As shown in Figure 10, it is obvious that the performance of ARA AC-13 far better. Its rutting resistance is better than that of other mixtures, and the cracking resistance of all the mixtures is not very different. 

The chemical composition of a polymer affects the performance of the asphalt mixtures. The ATR-FTIR spectra of the different modifiers (seen as Figure 2) show that there are different contents of alkanes, cycloalkanes, and other components in the polymers. These differences may lead to different mixture performances and aging sensitivity, which needs to be supported by further research. 

## 5. Conclusions and Recommendations

In this study, the effects of modifiers on the long-term performances of asphalt mixtures are evaluated. Based on the previous analyses and discussion, the following conclusions can be drawn.

(1) The rutting resistance of all asphalt mixtures changed into a V-shape as the aging progressed. From the unaged stage to the long-term aging stage (5 days), the shear strength decreased gradually. While after the long-term aging stage (5 days), the shear strength increased gradually.
(2) Both FI and *CT_index_* gradually declined with the increasing of the aging degree, indicating that aging weakened the crack resistance of the asphalt mixtures.
(3) Different polymer modifiers showed different sensitivities to aging. The long-term rutting resistance of ARA AC-13 was the best, and the long-term cracking resistance of ARA AC, HMM AC, and HVM AC showed no significant differences. In this study, the effect of ARA on the long-term performance of asphalt mixtures was more significant.

In future studies, other long-term properties of asphalt mixtures, such as water stability and fatigue properties, need to be further tested. In addition to this, although we recognize that the chemical composition of a polymer affects the long-term performance of asphalt mixtures, the relationship between the two has not been fully explored, and the effect of aging on the chemical composition of the polymer has not been fully investigated. These are directions for future research.

## Figures and Tables

**Table 1:** Properties of SBS asphalt.

Index	Unit	Value	Test Method
Penetration at 25 °C	0.1 mm	71	JTG E20 T0604
Penetration Index	-	0.5	JTG E20 T0604
Ductility at 5 °C	cm	48	JTG E20 T0605
Softening point	°C	64	JTG E20 T0606
Viscosity at 135 °C	Pa·s	1.8	JTG E20 T0625

**Table 2:** Properties of modifiers.

Items	Results of Various Mofifiers		
	HVM	HMM	ARA
Exterior	Black granules	Black granules	Black granules
Melt index (g/10 min)	6–12	5–11	5–11
Softening point (°C)	160–175	120–130	120–150
Dry mix dispersibility	No residue	No residue	No residue
Modifer content (%)	≥95	≥95	≥95

**Table 3:** Aggregate properties.

Aggregate Size (mm)	10–15	5–10	3–5	0–3
Bulk relative density	2.753	2.746	2.721	2.635
Apparent relative gravity	2.776	2.778	2.768	2.695
Water absorption (%)	0.30	0.42	0.62	0.84

**Table 4:** Volume parameters of AC-13.

Items	Optimal Asphalt Content (OAC) (%)	Voids Volume (VV) (%)	Voids in the Minreal Aggregate (VMA) (%)	Voids Filled with Asphalt (VFA) (%)
Values	4.67	4.1	14.2	71.1
Specification	-	3~6	≮14.0	65~75

## Data Availability

The data presented in this study are available on request from the corresponding author.
